# Explicit and implicit spatial mapping of face age

**DOI:** 10.1007/s00426-026-02256-y

**Published:** 2026-03-03

**Authors:** Mario Dalmaso, Stefano Pileggi, Mauro Murgia, Michele Vicovaro

**Affiliations:** 1 Department of Developmental and Social Psychology, University of Padova, Via Venezia 8, Padova, 35131 Italy; 2 Department of Life Sciences, University of Trieste, Trieste, Italy; 3 Department of General Psychology, University of Padova, Padova, Italy

## Abstract

Time is often conceptualised in spatial terms. For example, concepts such as ‘before’ and ‘after’ are frequently mapped onto the left and right sides of space, respectively. Importantly, such spatial mapping typically disappears when time is processed as a task-irrelevant dimension. In the present study, we further investigated this phenomenon using highly salient and socially relevant stimuli: human faces varying in age. We conducted two experiments using the same paradigm, in which the age of a female and a male face was processed either explicitly (Experiment 1; *N* = 120, 50% females) or implicitly (Experiment 2; *N* = 120, 50% females). In Experiment 1, participants categorised the age of a centrally-presented target face as younger or older than a reference face by pressing left or right keys. In Experiment 2, participants categorised the sex of the face. Both experiments provided evidence consistent with a left-to-right spatial mapping of face age, such that younger faces tended to be associated with the left and older faces with the right, although this effect was more pronounced when age was explicitly processed (Experiment 1). Experiment 2 also revealed a spatial mapping for the sex of the face. Taken together, these findings suggest that human faces are powerful stimuli capable of eliciting spatial representations not only of time, but also of other socially meaningful dimensions, such as sex, likely due to their salience and central role in social perception.

**Supplementary Information:**

The online version contains supplementary material available at 10.1007/s00426-026-02256-y.

## Introduction

Humans tend to spatially represent time along a left-to-right axis, with past events and short durations on the left, and future events and long durations on the right, a pattern especially prominent in Western cultures and influenced by reading and writing direction (Vallesi et al., 2009; Pitt & Casasanto, 2020). This spatial mapping is supported by both behavioural and neuroscientific evidence (Bonato et al., 2012). A clear behavioural example is the Spatial-Temporal Association of Response Codes (STEARC) effect, whereby participants respond faster to past events or short durations with a left-side key, and to future events or long durations with a right-side key (Ishihara et al., 2008). This phenomenon resembles the Spatial-Numerical Association of Response Codes (SNARC) effect, in which smaller numbers are associated with left-side responses and larger numbers with right-side responses (Dehaene et al., 1993).

Most studies exploring the STEARC effect have used temporal stimuli varying along perceptual (e.g., duration of sounds or visual stimuli; Ishihara et al., 2008; Vallesi et al., 2009) or conceptual dimensions (e.g., words like ‘yesterday’ or ‘tomorrow’; Santiago et al., 2007; Scozia et al., 2024; images showing temporal progressions; Kolesari & Carlson, 2018; Xiao et al., 2018). A consistent finding is that the STEARC effect would arise only when temporal information is task-relevant (see von Sobbe et al., 2019, for a meta-analysis). For example, it emerged when participants judged the timing of a sound but not its timbre (Mariconda et al., 2022), or the duration of a visual stimulus but not its shape (Dalmaso et al., 2023b). These results indicate that the STEARC effect depends on task demands, in contrast to the SNARC effect, which appears even when numerical magnitude is irrelevant, such as during parity judgments (Dehaene et al., 1993).

Recent studies have explored the STEARC effect in socially relevant domains, such as face age (Dalmaso & Vicovaro, 2021; Dalmaso et al., 2023a), a key social cue in face perception (e.g., Dalmaso et al., 2025; Jack & Schyns, 2015; Montepare & Zebrowitz, 1998) capable of influencing various cognitive and perceptual processes during social interactions, including attention allocation, emotional response, and social judgment (e.g., Dalmaso et al., 2020; Hayes et al., 2020; Rhodes & Anastasi, 2012). In Dalmaso et al. (2023a, Experiment 1), participants judged whether a target male face was younger or older than a reference face by pressing left or right keys. Responses were faster with a younger-left/older-right mapping, consistent with the STEARC effect. A distance effect also emerged, with faster responses for greater age differences. These findings indicate that face age can be spatially mapped along a left-to-right timeline, similar to other temporal dimensions.

The present study builds upon Dalmaso et al. (2023a) by investigating whether face age is automatically mapped onto the horizontal axis even when it is not explicitly relevant to the task. Two experiments were conducted. The first experiment aimed to replicate and extend Dalmaso et al. (2023a; Experiment 1) by including both a male and a female identity, requiring participants to classify a target face (regardless of sex) as younger or older than a reference face. In line with Dalmaso et al. (2023a; Experiment 1), we expected a left-to-right spatial mapping of face age and a distance effect. The second experiment employed the same paradigm but required participants to classify the target face as either male or female, making face age a task-irrelevant dimension. Here, two alternative hypotheses were considered. On the one hand, the first predicted that no spatial mapping of face age would be observed, based on prior findings showing that the STEARC effect emerged only when the temporal dimension of symbolic stimuli was task-relevant (Dalmaso et al., 2023a; Mariconda et al., 2022). On the other hand, a second, more tentative hypothesis predicted that a STEARC effect might still emerge, possibly due to the inherently high relevance of face age in human social perception and behaviour (Dalmaso et al., 2020; Hayes et al., 2020; Rhodes & Anastasi, 2012). Such relevance is, by definition, absent in more abstract, non-social dimensions, such as the duration of visual or auditory stimuli used in previous studies (Dalmaso et al., 2023b; Mariconda et al., 2022).

## Experiment 1: face age as an explicit dimension

### Participants

The sample size was established a priori. As we planned to analyse the data through generalised linear mixed-effects models, including both items and subjects as random effects (see the Results section), the sample size was defined following dedicated guidelines (Brysbaert & Stevens, 2018). These guidelines indicate collecting a minimum of 1600 trials per experimental condition (in designs with repeated measures) to achieve adequate statistical power. Because we planned to collect 30 trials per condition for each participant, a minimum of approximately 54 participants was required. Given that previous studies have shown that sex could play a role in face categorisation (e.g., Verdichevski & Steeves, 2013) and may also be spatially represented (Calvente et al., 2023), we included an equal number of male and female participants and face identities to ensure that our findings were not biased by sex-related factors. Accordingly, both participant sex and face sex were included in the analyses to comprehensively assess their potential influence on the data, even though our hypotheses focused on face age, which was considered the primary variable within our experimental context. We therefore collected data from 60 female and 60 male individuals (*Mean age* = 23.2 years; *SD* = 1.52). We recruited slightly more than 54 participants per group, anticipating data loss due to incorrect or missing responses (see the Results section). Participants were recruited on Prolific (https://www.prolific.com/; £7.60 hourly rate). All the participants were White Italians speaking Italian as their primary language. Manual preference was assessed using the short form of the Edinburgh Handedness Inventory (EHI; Veale, 2014), which provides a score on a continuous scale from − 100 to + 100 (i.e., strong preference for either the left or the right hand, respectively). The mean EHI score of this sample was + 68.5 (*SD* = 46.1; range = − 100 to + 100). According to the EHI, nine participants were classified as left-handed, six as mixed-handed, and 105 as right-handed.

## Apparatus, stimuli, and procedure

**Fig. 1:**
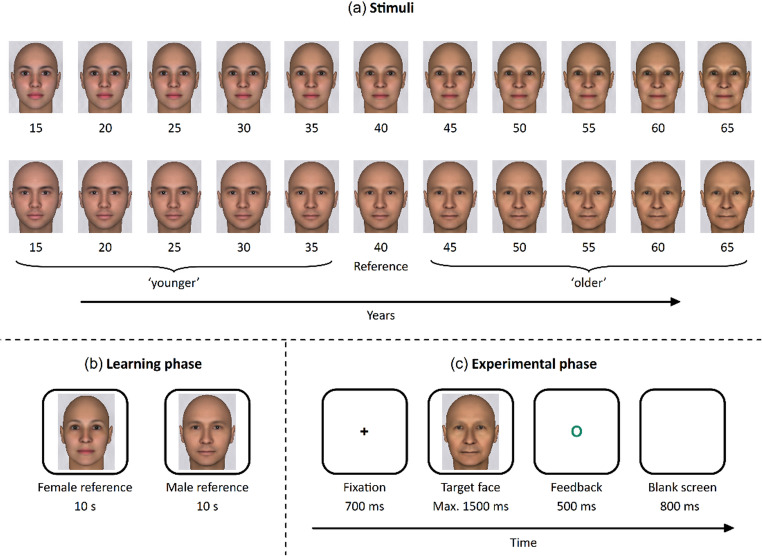
The female and male faces used in both experiments are shown in panel **A**. Participants first completed a learning phase (panel **B**), during which they observed each reference face (one female and one male) for 10 s. This was followed by the experimental phase (panel **C**), where they either determined whether the target face appeared younger or older than the reference (Experiment 1) or identified its sex as female or male (Experiment 2)

The experiment was programmed with PsychoPy (https://www.psychopy.org/) and delivered online through Pavlovia (https://pavlovia.org/). The study utilised a set of 22 faces (300 × 400 pixels), based on a female and a male model with neutral expressions (see Fig. 1). The male model was also employed in Dalmaso et al. (2023a). The two models depicted White individuals, chosen to match the ethnicity of the participants and avoid potential group biases in spatial mapping (e.g., Dalmaso et al., 2023c; Presaghi & Rullo, 2018). All faces, spanning ages from 15 to 65 in 5-year increments, were generated using FaceGen Modeller (https://facegen.com/), a software designed to create realistic and highly controlled facial stimuli. To prevent any perceptual inconsistencies, the images were standardised for luminance and spatial frequency using the SHINE_color toolbox in MATLAB (Dal Ben, 2023). The faces of the two models aged 40 years served as the reference point (see the next section), while the remaining images represented ages either younger or older than this reference.

The experimental procedure was similar to that employed by Dalmaso et al. (2023a). First, in the learning phase (see Fig. 1), participants viewed one of the two reference faces, displayed in the centre of the screen for 10 s, accompanied by the text ‘This person is 40 years old’ positioned above the image. Then, the second reference face was displayed in the same manner. The two reference faces were presented in a random order. Following this, the experimental phase began. Each trial started with a black fixation cross (Arial font; height: 0.085 of normalised units) presented at the centre of the screen for 700 ms. This was followed by the appearance of a target face. Participants had to respond within a 1500-ms time window. Visual feedback was displayed for 500 ms, indicating the outcome of their response. A green ‘O’ indicated a correct answer, a red ‘X’ indicated an error, and the message ‘Too slow’ was shown if no response was detected. An 800 ms blank screen separated trials. The task required participants to categorise the target face as younger or older than 40 years as quickly and accurately as possible. Responses were made by pressing one of two keys on the keyboard: the ‘D’ key with the left index finger or the ‘K’ key with the right index finger. The experiment consisted of two main blocks of 120 trials each (240 experimental trials in total), which were preceded by a practice block of 10 trials (20 practice trials in total). Each target face was presented an equal number of times in random order within each block. The response mapping alternated between blocks. In the ‘congruent’ block, younger faces were assigned to the left key and older faces to the right key. In the ‘incongruent’ block, the mapping was reversed. The order of the blocks was counterbalanced across participants. In summary, the 240 experimental trials were derived from the following design: 2 blocks (congruent vs. incongruent) × 2 face sexes (male vs. female) × 10 face ages (15 to 65 years in 5-year increments, excluding age 40) × 6 repetitions. At the end of the task, participants completed a computerised version of the EHI (Veale, 2014).

## Results

Missed responses were rare (1.41% of trials) and were discarded from further analysis. Incorrect responses (10.22% of trials) were also excluded and analysed separately. Trials with a correct response were cleaned by removing reaction times (RTs) shorter than 100 ms or greater than 3 SDs above the mean, calculated separately for each participant and experimental condition (1.06% of trials).

## STEARC effect

Face age was treated as a dichotomous (vs. continuous) predictor for two reasons. First, RTs did not vary as a continuous monotonic function of face age (see the Supplementary Materials, Figure S1, Panel A; this aspect is further addressed in the discussion of the distance effect below). Second, previous studies have shown that, in explicit classification tasks, SNARC and SNARC-like effects tend to follow a step-like rather than a linear pattern (Gevers et al., 2006), a conclusion that was also supported by our data (see the Supplementary Materials, Figure S1, Panel B). RTs were therefore analysed using generalised linear mixed-effects models (R package *lme4*; Bates et al., 2015) with an identity link function for a Gamma-distributed response variable (Lo & Andrews, 2015). The fixed-effects structure included all main effects and interactions of the four experimental factors: face age (younger vs. older than the reference), response side (left vs. right), participant sex, and face sex. To identify the optimal random-effects structure, we incrementally added random components to the fixed-effects model, beginning with by-participant or by-item random intercepts and progressing to models that included by-participant and by-item random intercepts and by-participant random slopes for face age, response side, and face sex. Please note that ‘item’ here refers to the specific face stimulus presented in a given trial (i.e., 20 items in total, corresponding to 10 ages for each face sex). These models were then compared according to the Akaike Information Criterion (AIC) using the R package *MuMIn* (Bartoń, 2023). The best-fitting model included, as random effects, the by-participant intercept, the by-participant slope for face age, and the by-item intercept. A Type 3 Wald chi-square test was then conducted on this model to assess the significance of the predictors and their interactions (R package *car*; Fox & Weisberg, 2019).

For the sake of brevity, here and in the following sections, all results not related to our primary interest (i.e., a spatial mapping of face age) are reported in the Supplementary Materials. In addition, Table 1 in the Supplementary Materials provides an additional overview of the findings. In summary, the key secondary result showed that, overall, RTs associated with younger faces were shorter for female than for male identities, whereas the opposite pattern was observed for older faces, namely shorter RTs for male than for female identities (see the Supplementary Materials, Section 1A). This may reflect an implicit association linking youth with femininity and older age with masculinity (see also, e.g., Fitousi, 2021), an interpretation further explored in the General Discussion.

**Fig. 2:**
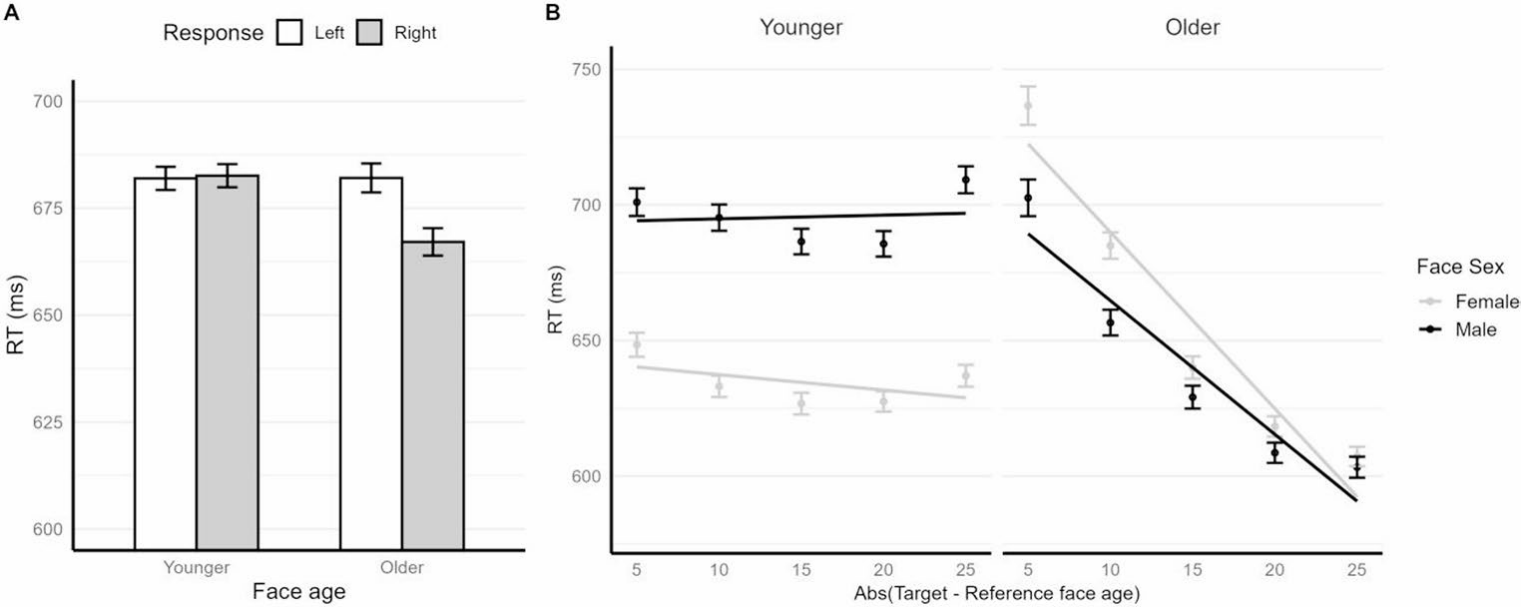
Main results of Experiment 1. Panel** A** displays the mean RTs as a function of response side and face age, while panel** B** illustrates the relationship between RTs and the absolute difference between the target and reference face age (separately for face age and sex). Error bars represent the standard error of the mean

As for the main hypothesis, the response side × face age interaction was significant, *χ*^2^(1) = 29.38, *p* < .001, thus confirming the presence of a STEARC effect. Further comparisons revealed, for older faces, longer RTs (*p* < .001) for the left-side key (*M* = 682 ms, *SE* = 2.49) than the right-side key (*M* = 667 ms, *SE* = 2.47), while no significant difference was found for younger faces, where RTs for the left-side key (*M* = 682 ms, *SE* = 2.74) and the right-side key (*M* = 683 ms, *SE* = 2.86) were similar (*p* = .759; see also Fig. 2A). Importantly, the response side × face age interaction was not further qualified by a three-way interaction involving face sex, *χ*^2^(1) = 0.019, *p* = .89, or participant sex, *χ*^2^(1) = 0.84, *p* = .361. The four-way interaction was also non-significant, *χ*^2^(1) = 0.53, *p* = .468, indicating that the STEARC effect was consistent across both face sex and participant sex (see the Supplementary Materials, Section 1B).

Accuracy data were analysed using the same approach as RTs but, in this case, we used generalised linear mixed-effects models with a logit link function for a Binomial-distributed response variable. The best-fitting model included, as random effects, the by-participant and by-item random intercepts, as well as the by-participant random slopes for face age and face sex. The response side × face age interaction was significant, *χ*^2^(1) = 5.91, *p* = .015. Further comparisons revealed that, for younger faces, right-side responses were slightly more accurate (*M* = 0.947, *SE* = 0.014) than left-side responses (*M* = 0.938, *SE* = 0.016, *p* = .020). For older faces, accuracy differences between left-side responses (*M* = 0.929, *SE* = 0.018) and right-side responses (*M* = 0.925, *SE* = 0.019, *p* = .314) were non-significant. The direction of this interaction contrasts with that observed for RTs, suggesting a possible speed–accuracy trade-off. However, the small overall differences in accuracy across face age and response side combinations (never exceeding 2.2% points) support the robustness of the STEARC effect in RTs. No additional evidence of a speed–accuracy trade-off emerged from the remaining accuracy results, which largely mirrored those of the RT analysis. Briefly, for younger faces, accuracy was higher for female than for male faces, whereas for older faces the opposite pattern emerged, with higher accuracy for male than for female faces (see the Supplementary Materials, Sect. 1C). This supports the young-female / old-male association also observed in the RT analysis.

## Distance effect

RTs were analysed using generalised linear mixed-effects models with a Gamma-distributed response and an identity link. Fixed effects included all main effects and interactions between the absolute age difference from the reference face (5, 10, 15, 20, and 25 years; hereafter *distance*), face age (younger vs. older), face sex, and sex of participants. Random effects included intercepts for each participant and slopes for face age.

Effects not involving distance mirrored those observed in the STEARC RT analysis and are not discussed further. There was a significant effect of distance (*b* = − 5.90, *SE* = 0.217, *p* < .001), with RTs decreasing as the age difference between the target and reference face increased. This effect was modulated by face sex (*b* = 1.547, *SE* = 0.192, *p* < .001) and, more strongly, by face age (*b* = 5.363, *SE* = 0.269, *p* < .001). Importantly, distance, face age, and face sex also interacted (*b* = 0.654, *SE* = 0.274, *p* = .016), thus inviting separate analyses for younger and older faces (see also Fig. 2B). In brief, these analyses revealed a clear distance effect for older faces, but a negligible effect for younger faces (see the Supplementary Materials, Section 1D). For both age groups, the effect was slightly stronger for female than male faces. Although accuracy indicated that participants reliably distinguished younger and older faces from the reference, only older faces showed a progressive facilitation as age distance increased, suggesting that they were perceived as increasingly different from the reference. By contrast, the lack of a distance effect for younger faces suggests that they were likely perceived as relatively similar in age, and thus that perceived face age did not scale linearly with simulated age. Crucially, this does not undermine the STEARC results, as face age was treated dichotomously and specific face ages were modelled as random effects, ensuring valid interpretability.

## Discussion

The main results provided a conceptual replication of those reported by Dalmaso et al. (2023a; Experiment 1), confirming an interaction between response side and face age that was consistent with a left-to-right spatial representation of face age. Moreover, they indicated that this representation was consistent across both face sex and participant sex, whereas in Dalmaso et al. (2023a) only a single male identity was used, and participant sex was not considered. The distance-effect analysis showed that the ages of older faces were perceived in a quasi-linear manner, whereas the ages of younger faces were not clearly discriminated from one another, although they were still clearly distinguished from the reference face. This pattern may partly reflect the use of artificial face stimuli (please see the General Discussion). However, this does not affect the interpretability of the RT and accuracy results related to the STEARC effect reported here and in Dalmaso et al. (2023a).

## Experiment 2: face age as an implicit dimension

### Participants

As in Experiment 1, a new group of 120 White Italians speaking Italian as their primary language (*Mean age* = 24.5 years; *SD* = 2.14; 60 females and 60 males) was recruited on Prolific. The mean EHI score was + 62.9 (*SD* = 54.8; range = − 100 to + 100), and 12 participants were classified as left-handed, 11 as mixed-handed, and 97 as right-handed.

### Apparatus, stimuli, and procedure

Everything was identical to Experiment 1, with two exceptions: during the learning phase, participants were instructed to focus on the sex of the face (accompanied by the text ‘This person is female’ or ‘This person is male’, according to the sex of the depicted face), and during the experimental phase, they were asked to categorise the sex of the face. In one block, male faces were assigned to the left key and female faces to the right key, in the other block the mapping was reversed. The presentation order of the two blocks was counterbalanced across participants.

## Results

Data were handled and analysed as in Experiment 1. Missed responses (0.69% of trials) were excluded from further analysis. Incorrect responses (3.26%) were analysed separately. RT outliers (1.24%) were removed.

### STEARC effect

Consistent with Experiment 1, initial data exploration indicated that treating face age as a dichotomous (vs. continuous) predictor was more appropriate (see Figure S2 in the Supplementary Materials). The best-fitting model included, as random effects, the by-participant intercept, the by-participant slopes for response side and face sex, and the by-item intercept. All results not related to our primary interest (i.e., a spatial mapping of face age) are reported in the Supplementary Materials. In addition, Table 2 in the Supplementary Materials provides an additional overview of the findings. In summary, the key secondary result confirmed the association between face age and face sex already observed in Experiment 1: overall, RTs associated with younger faces were shorter for female than for male identities, whereas the opposite pattern was observed for older faces (i.e., shorter RTs for male than for female identities; see the Supplementary Materials, Section 2A).

**Fig. 3:**
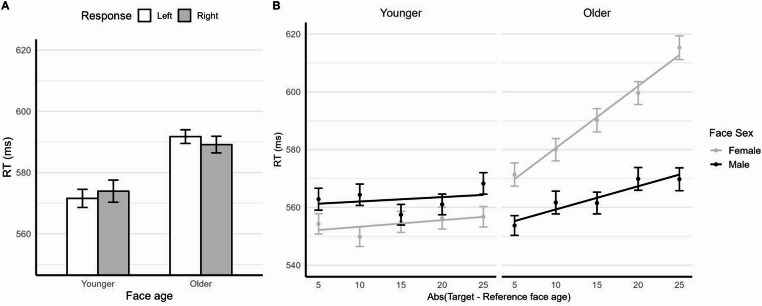
Panel **A** displays the mean RTs as a function of response side and face age, while panel **B** illustrates the relationship between RTs and the absolute difference between the target and reference face age (separately for face age and sex). Error bars represent the standard error of the mean

As for the main hypothesis, the analysis showed that the response side × face age interaction was significant, *χ*^2^(1) = 4.43, *p* = .035, thus indicating an implicit STEARC effect for face age. Further comparisons revealed, for older faces, non-significantly longer RTs (*p* = .338) for the left-side key (*M* = 592 ms, *SE* = 2.71) than the right-side key (*M* = 589 ms, *SE* = 2.73), and for younger faces, non-significantly longer RTs (*p* = .365) for the right-side key (*M* = 574 ms, *SE* = 2.26) than the left-side key (*M* = 572 ms, *SE* = 2.54; see also Fig. 3A). This interaction was not qualified by three-way interactions involving face sex, *χ*^2^(1) = 0.01, *p* = .756, or participant sex, *χ*^2^(1) = 0.87, *p* = .351, nor by the four-way interaction, *χ*^2^(1) ≈ 0.0, *p* = .979, thus indicating that, as in Experiment 1, the STEARC effect was consistent across both face sex and participant sex (see the Supplementary Materials, Section 2B).

**Fig. 4:**
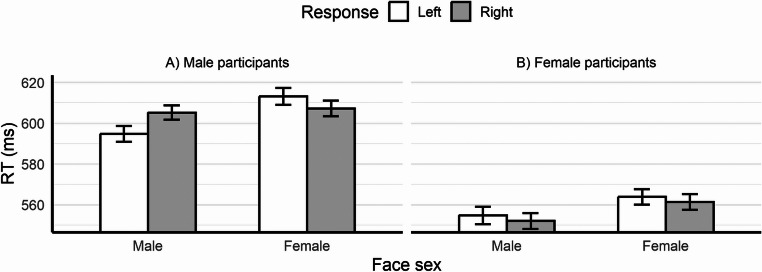
Mean RTs as a function of response side and face sex. Panel A displays the results for male participants, panel B displays the results for female participants. Error bars represent the standard error of the mean

Intriguingly, a spatial representation of face sex also emerged. The response side × face sex interaction was significant, *χ*^2^(1) = 11.45, *p* < .001, further qualified by the higher order response side × face sex × participant sex interaction, *χ*^2^(1) = 11.16, *p* < .001. The three-way interaction was further analysed by splitting the data for participant sex. For male participants, the main effects of response side and face sex were not statistically significant [*χ*^2^(1) = 0.51, *p* = .474 and *χ*^2^(1) = 3.36, *p* = .067, respectively], whereas the response side × face sex interaction was significant, *χ*^2^(1) = 20.71, *p* < .001 (see Fig. 4A). The interaction was analysed with further comparisons, which showed that for male faces, longer RTs (*p* = .013) emerged with the right-side key (*M* = 607 ms, *SE* = 5.41) than with the left-side key (*M* = 596 ms, *SE* = 5.14), whereas for female faces, RTs were non-significantly longer (*p* = .228) with the left-side key (*M* = 614 ms, *SE* = 4.14) than with the right-side key (*M* = 609 ms, *SE* = 4.03). This pattern of results is consistent with a SNARC-like effect for face sex, in which male faces are mapped to the left and female faces to the right. Interestingly, this effect was limited to male participants, indeed for female participants the response side × face sex interaction was not statistically significant, *χ*^2^(1) = 0.004, *p* = .952 (see also Fig. 4B), nor were the main effects of response side and face sex [*χ*^2^(1) = 0.34, *p* = .348 and *χ*^2^(1) = 3.52, *p* = .061, respectively; see the Supplementary Materials, Sect. 2C].

As for accuracy, the best-fitting model included, as random effects, the by-participant intercept, the by-participant slope for face age, and the by-item intercept. The main effects of face age, response side and face sex were statistically significant (see the Supplementary Materials, Section 2D). The response side × face age interaction was not statistically significant, *χ*^2^(1) = 2.34, *p* = .126, nor were the other interactions (*p*s ≥ 0.098).

### Distance effect

The distance effect is typically observed only for task-relevant dimensions. In this case, it could not be calculated for face sex because this variable is dichotomous. However, for completeness, we calculated it for the task-irrelevant dimension of face age, using the same approach adopted for the analysis of the distance effect in Experiment 1.

Effects not involving distance mirrored those observed in the STEARC RT analysis and are not discussed further. There was a significant effect of distance (*b* = 2.371, *SE* = 0.196, *p* < .001), with RTs increasing as the age difference between the target and reference face increased (i.e., an inverted distance effect). This effect was modulated by face sex (*b* = 1.571, *SE* = 0.225, *p* < .001) and by face age (*b* = 2.206, *SE* = 0.194, *p* < .001). Furthermore, the interaction between distance, face age, and face sex was strong and significant (*b* = 7.273, *SE* = 1.431, *p* < .001). To examine this interaction, we ran separate analyses for younger and older faces (see the Supplementary Materials, Section 2E; see also Fig. 3B). In brief, these analyses showed no distance effect for younger faces and a clear inverted distance effect for older faces. For older faces, the inverted distance effect was stronger for female than for male faces. This pattern may reflect a general tendency to associate female faces with younger age and male faces with older age (see also, e.g., Fitousi, 2021), which could explain the linear increase in RT differences between female and male faces as face age increased. It may also reflect increased difficulty in classifying face sex at older ages, which could explain the RT increase for both female and male faces with increasing face age^1^. For younger faces, the absence of distance effects and of interactions with face sex is consistent with the hypothesis that these faces were perceived as similar in age.

## Discussion

As in Experiment 1, the interaction between response side and face age was consistent with the possibility that a STEARC effect for face age may emerge even when this dimension is task-irrelevant, potentially reflecting a relatively automatic mapping onto the horizontal axis. The magnitude of this implicit effect was clearly smaller than the explicit STEARC effect observed in Experiment 1 (see Figs. 2A and 3A) but, importantly, mean RTs across conditions defined by response side and face age were in the expected direction. Moreover, evidence of an inverted distance effect emerged for older faces but not for younger faces. Finally, a SNARC-like effect also emerged for the explicit dimension of face sex, but only among male participants. These findings will be further examined in the General Discussion.

### General discussion

This study investigated whether face age was spatially mapped along a left-to-right mental timeline, both when explicitly processed (Experiment 1) and when task-irrelevant (Experiment 2). In both experiments, the main result related to our primary hypothesis (i.e., a spatial mapping of face age) was an interaction between response side and face age, which may be interpreted as being consistent with a left-to-right spatial representation of face age, in line with previous work (see Dalmaso et al., 2023a). This effect was consistent across face and participant sex, supporting the generalisability of the phenomenon.

A key finding of this study was that the interaction between response side and face age was also observed in Experiment 2, where age was not a task-relevant dimension, as participants were asked to classify the sex of the face. This pattern is consistent with the possibility that face age may be mapped onto space even in the absence of explicit age processing. This result contrasts with recent studies on the STEARC effect assessed at an implicit level (e.g., Dalmaso et al., 2023b; Mariconda et al., 2022), which found that no spatial-temporal association emerged when the temporal dimension of a stimulus was irrelevant (see also von Sobbe et al., 2019)^2^. One possible explanation for the results reported here concerns the fundamental role that faces play in human perception and social interactions, with age being one of their most salient characteristics (e.g., Jack & Schyns, 2015; Montepare & Zebrowitz, 1998). By contrast, in the studies by Dalmaso et al. (2023b); Mariconda et al. (2022), participants were exposed to non-social stimuli, such as geometric shapes (e.g., a square or a diamond) or sounds with different timbres (e.g., a metronome or electronic sound), and therefore, it was reasonable to assume that their temporal dimension was likely less pervasive and less intrinsically relevant to the participants compared to the temporal dimension manipulated in the present study. Nevertheless, this interpretation should be considered speculative. Future studies should compare face age with non-social stimuli that can also convey temporal information (e.g., trees at different stages of growth, puppies vs. adult animals), to clarify whether the persistence of the effect is specific to human faces or reflects age saliency more generally.

From a quantitative perspective, it is important to note that the implicit STEARC effect observed in Experiment 2 was small, particularly when compared to the robust explicit effect found in Experiment 1 (see Figs. 2A and 3A). This invites two considerations. First, given their small size, implicit STEARC effects (and, more broadly, implicit SNARC-like effects in general) may only emerge with large sample sizes. In this respect, our sample size (*N* = 120) was approximately four times larger than that used in typical STEARC studies (see Dalmaso et al., 2023b; Mariconda et al., 2022). Second, although the spatial representation of face age appears sufficiently strong and automatic to emerge even when task-irrelevant, it is clearly more pronounced when age is processed explicitly. This pattern supports the view that spatial representations of non-numerical quantities are less robust and automatic than those of numerical information (Macnamara et al., 2018).

Related to the previous point, it is also important to note that, although the critical interaction between response side and face age emerged reliably in both experiments, follow-up comparisons revealed an asymmetric pattern. For example, in Experiment 1, the comparison between left-side and right-side responses was significant for older but not for younger faces. Such asymmetries, however, should not be considered unusual. Similar unbalanced effects have been reported for several dimensions, including loudness, time, speed, weight, size, economic value, and face age (e.g., Chang & Cho, 2015; Dalmaso et al., 2023a, b, 2026; Dalmaso & Vicovaro, 2019; Giuliani et al., 2024; Mariconda et al., 2024; Ren et al., 2011; Vicovaro et al., 2025). These asymmetries appear to occur more frequently for non-numerical than for numerical magnitudes (but see, e.g., Di Rosa et al., 2017) and may be related to a more direct or precise spatial mapping of numerical information compared to non-numerical dimensions (see also Giuliani et al., 2021). They may also be related to the fact that the large majority of participants were right-handed, which could amplify the RT difference between the left and right keys for stimuli mapped to the right (i.e., older faces) while simultaneously reducing the difference for stimuli mapped to the left (i.e., younger faces). Nevertheless, the mechanisms underlying these unbalanced patterns remain poorly understood, and future research will be necessary to systematically investigate their origins.

A limitation of the present study concerns the use of artificially generated stimuli, which limits the ecological validity of the results. To our knowledge, no database standardised for psychological research currently offers photographs of the same individuals at different ages, which makes the use of such stimuli unfeasible. For this reason, we relied on artificially rendered faces. Nevertheless, the software we employed is widely used in face perception research and has been successfully adopted to manipulate several dimensions, such as trustworthiness or dominance (see, e.g., Todorov et al., 2008). Although this approach cannot fully capture the richness of natural facial aging, it offers a high level of experimental control and allows for the systematic manipulation of age-related cues. Although we did not collect a direct measure of how participants perceived the different ages of the faces, the high level of accuracy observed in the age classification task of Experiment 1 provides indirect evidence that age differences were successfully encoded. At the same time, the lack of a distance effect for young faces indicates that these faces were probably perceived as relatively similar in age, and thus that perceived face age did not scale linearly with simulated age. Notably, after our data collection had been completed, we identified a converging pattern in the literature. Specifically, Short et al. (2019; Experiment 1), employing morphed photographs of real human faces (depicting a male identity), observed reduced sensitivity in age judgments for the young-adult segment of an age continuum relative to older segments. This convergence suggests that limited differentiation among younger faces may reflect a more general characteristic of face age perception, rather than a specific artefact related to our stimuli. Nevertheless, future studies investigating the spatial mapping of face age (using either artificial or real faces placed along a temporal continuum), should also include direct measures of perceived age for each stimulus, to obtain a more complete picture of how face age is represented and to better inform the interpretation of spatial mapping effects.

The rest of the General Discussion addresses additional findings on the performance of male and female participants in processing face sex, beyond the STEARC effect. First, in Experiment 1, participants (regardless of their sex) were, overall, better at categorising female faces as younger and male faces as older (see the Supplementary Materials, sections 1A and C). This pattern may reflect social stereotypes linking youth to women and aging to men, shaped by cultural norms and perceptual biases (e.g., Fitousi, 2021). A similar advantage for younger female and older male faces also appeared in Experiment 2 (see the Supplementary Materials, sections 2A and D), supporting the robustness of this effect. Second, in Experiment 2, classifying face sex was more difficult for older faces, likely due to age-related changes in sexually dimorphic features that make sex identification less reliable (e.g., Velemínská et al., 2022). Third, and most importantly, Experiment 2 also revealed a SNARC-like effect for face sex, observed only in male participants: male and female faces were mapped to the left and right, respectively. This pattern does not align with the findings of Calvente et al. (2023; Experiment 1), who observed the opposite mapping (i.e., female-left/male-right), regardless of participant sex. However, methodological differences between the two studies prevent direct comparison and limit any definitive conclusions. In particular, in Calvente et al. (2023; Experiment 1), participants were presented with photographs of 40 real identities but also with female and male name stimuli. Future research should aim to clarify the underlying mechanisms and moderating factors that govern the spatial representation of face sex.

In sum, this study provided further evidence for the spatial representation of age in face perception, extending previous findings (e.g., Dalmaso & Vicovaro, 2019; Dalmaso et al., 2023a). It also contributed to a growing body of research on the STEARC effect and its potential implications for understanding how age influences social perception.

## Supplementary Information

Below is the link to the electronic supplementary material.

Supplementary Material 1

## Data Availability

Data, materials, and codes are available on OSF: (https://doi.org/10.17605/OSF.IO/HM4PF). The study was not preregistered.

## Footnotes

1 For the sake of clarity, we note that any apparent discrepancies between Experiments 1 and 2 in the distance-effect results are likely attributable to the different task demands, with face age being task-relevant (and face sex task-irrelevant) in Experiment 1, and face sex being task-relevant (and face age task-irrelevant) in Experiment 2.

2 For the sake of completeness, it is worth noting that one recent work (Topić et al., 2022) did observe an implicit spatial representation of time. However, as Topić et al. (2022) used a visual search task and the space-time association emerged along the vertical and diagonal axes, a direct comparison with the present set of experiments is not straightforward.

## Declarations

### Open practices

Data, materials, and codes are available on OSF: https://doi.org/10.17605/OSF.IO/HM4PF. The study was not preregistered.
