# Supplementary material for: Explicit and implicit spatial mapping of face age
Source: Psychol Res. 2026 Mar 3;90(2):50. doi: 10.1007/s00426-026-02256-y (PMC12956975; doi:10.1007/s00426-026-02256-y)
Supplement: Supplementary file 1 — Supplementary Material 1 [file 426_2026_2256_MOESM1_ESM.docx]

**Explicit and implicit spatial mapping of face age**

Mario Dalmaso, Stefano Pileggi, Mauro Murgia, & Michele Vicovaro

**Supplementary Materials**

**Experiment 1**


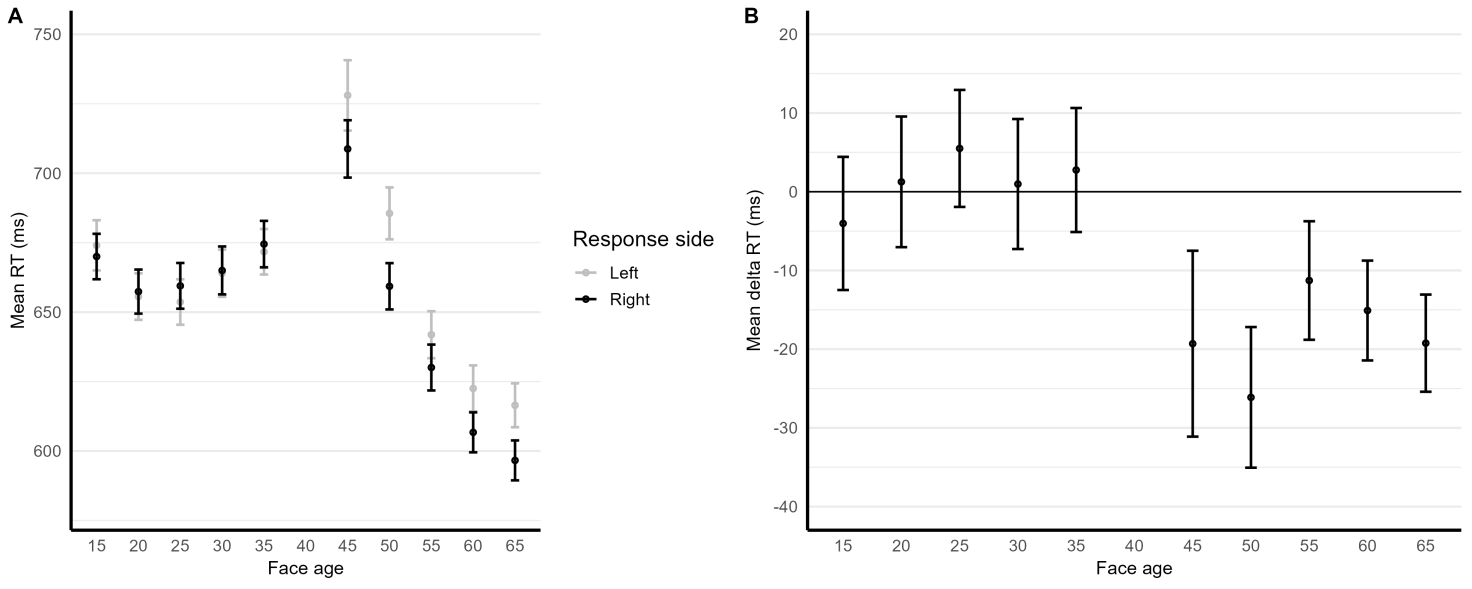


**Figure S1**. Panel A shows mean RTs, averaged across participants, as a function of face age and response side. RTs do not vary as a simple monotonic function of face age. Panel B shows the mean difference between RTs for the right and left response keys (delta RT), averaged across participants, as a function of face age. Individual mean RTs for each response side were computed by averaging across repetitions of each face age. As shown in Panel B, delta RT—indexing the strength of the STEARC effect for face age—exhibits a step-like rather than continuous pattern. Specifically, a relatively large difference is observed between younger (15–35 years) and older faces (45–65 years), whereas no clear linear trend emerges within each age range. Overall, the patterns observed in Panels A and B justify treating face age as a dichotomous (younger vs. older), rather than continuous, predictor in the main STEARC analysis. In both panels, error bars represent the standard error of the mean.

*1A*

The analysis revealed significant main effects of face age, *χ*^2^(1) = 5.07, *p* = .024, with longer RTs for younger faces (*M* = 682 ms, *SE* = 2.60) than for older faces (*M* = 675 ms, *SE* = 2.60); response side, *χ*^2^(1) = 22.48, *p* < .001, with longer RTs for responses with the left-side key (*M* = 682 ms, *SE* = 1.88) than the right-side key (*M* = 675 ms, *SE* = 1.90); and face sex, *χ*^2^(1) = 25.11, *p* < .001, with longer RTs for male faces (*M* = 687 ms, *SE* = 2.59) than for female faces (*M* = 670 ms, *SE* = 2.23). Participant sex was non-significant, *χ*^2^(1) = 1.71, *p* = .191. A significant response side × participant sex interaction emerged, *χ*^2^(1) = 29.38, *p* = .002, with female participants being significantly slower (*p* < .001) with the left-side key (*M* = 686 ms, *SE* = 2.62) than the right-side key (*M* = 675 ms, *SE* = 2.60), while no significant difference was found for male participants, who were only slightly slower with the left-side key (*M* = 678 ms, *SE* = 2.40) than with the right-side key (*M* = 675 ms, *SE* = 2.52, *p* = .187). The face age × face sex interaction was significant, *χ*^2^(1) = 208.9, *p* < .001, as well as the face sex × participant sex interaction, *χ*^2^(1) = 12.93, *p* < .001. These interactions were further qualified by the higher-order face age × face sex × participant sex interaction, *χ*^2^(1) = 64.13, *p* < .001. No other significant interactions emerged (*p*s ≥ .056). The significant three-way interaction was further analysed by splitting the data for participant sex. For male participants, the two main effects and the face age × face sex interaction were significant (*p*s < .001). The interaction was analysed with further comparisons, which showed that for younger faces, longer RTs (*p* < .001) emerged for male faces (*M* = 698 ms, *SE* = 4.89) than for female faces (*M* = 657 ms, *SE* = 3.63), whereas for older faces, longer RTs (*p* < .001) emerged for female faces (*M* = 678 ms, *SE* = 2.71) than for male faces (*M* = 663 ms, *SE* = 3.47). The same pattern also emerged for female participants, with the two main effects and the interaction being significant (*p*s < .001). Also in this case, for younger faces, longer RTs (*p* < .001) emerged for male faces (*M* = 723 ms, *SE* = 5.02) than for female faces (*M* = 650 ms, *SE* = 3.65), whereas, for older faces, longer RTs (*p* < .001) emerged for female faces (*M* = 688 ms, *SE* = 2.79) than for male faces (*M* = 659 ms, *SE* = 3.70); notably, these differences were greater than the ones observed among male participants, thus explaining the three-way interaction.

*1B*

As recommended by one reviewer, we also conducted exploratory analyses restricted to right-handed participants, given that manual preference may play a role in the spatial representation of concepts (e.g., Casasanto, 2009). All main results were consistent with those obtained from the full sample (see the analysis file on OSF).

*1C*

The main effects were not statistically significant (*p*s ≥ .254). The face sex × participant sex interaction was significant, *χ*^2^(1) = 5.04, *p* = .025, but this was further qualified by the higher-order face age × face sex × participant sex interaction, *χ*^2^(1) = 18.16, *p* < .001. No other significant interactions emerged (*p*s ≥ .075). The significant three-way interaction was further analysed by splitting the data for participant sex. For male participants, the two main effects and the face age × face sex interaction were not significant (*p*s ≥ .205). Mean accuracy data showed that for younger faces, higher accuracy emerged for female faces (*M* = .956, *SE* = 0.017) than for male faces (*M* = .913, *SE* = 0.031), whereas for older faces, higher accuracy emerged for male faces (*M* = .931, *SE* = 0.025) than for female faces (*M* = .913, *SE* = 0.031). The same pattern also emerged for female participants, however in this case the two main effects were not significant (*p*s ≥ .143), whereas the interaction was significant, *χ*^2^(1) = 5.11, *p* = .024. Also in this case, for younger faces, higher accuracy (*p* = .009) emerged for female faces (*M* = 0.974, *SE* = 0.011) than for male faces (*M* = 0.898, *SE* = 0.037), whereas, for older faces, higher accuracy emerged for male faces (*M* =.940, *SE* = 0.023) than for female faces (*M* =.920, *SE* = 0.030), although the difference was not statistically significant (*p* = .581); notably, these differences were greater than the ones observed among male participants, thus explaining the three-way interaction. This pattern of results mirrors the RT analysis.

In addition, as recommended by one reviewer, we ran exploratory analyses restricted to right-handed participants which yielded results that closely mirrored those obtained from the full sample (see the analysis file available on OSF).

*1D*

For younger faces, the best-fitting model included distance and face sex as fixed effects, and by-participant intercepts and slopes for face sex as random effects. The interaction between distance and face sex was significant (*b* = 0.664, *SE* = 0.264, *p* = .011), reflecting a small but significant distance effect for female faces (*b* = –0.544, *SE* = 0.190, *p* = .004) and no distance effect for male faces (*b* = 0.120, *SE* = 0.216, *p* = .58). For older faces, the best-fitting model included distance and face sex as fixed effects, and by-participant intercepts and slopes for distance as random effects. The interaction between distance and face sex was significant (*b* = 1.425, *SE* = 0.371, *p* < .001), reflecting a stronger distance effect for female faces (*b* = –6.100, *SE* = 0.281, *p* < .001) than for male faces (*b* = –4.680, *SE* = 0.351, *p* < .001).

**Table 1.** Main results observed in Experiment 1.

| *Effect* | *Test* | *df* | *Statistic* | *p* |
| --- | --- | --- | --- | --- |
| Response side × Face age (RTs) | χ² | 1 | 29.38 | < .001 |
| Response side × Face age × Face sex (RTs) | χ² | 1 | 0.019 | .890 |
| Response side × Face age × Participant sex (RTs) | χ² | 1 | 0.84 | .361 |
| Response side × Face age × Face sex × Participant sex (RTs) | χ² | 1 | 0.53 | .468 |
| Response side × Face age (Accuracy) | χ² | 1 | 5.91 | .015 |
| Distance (RTs) | b | – | -5.90 (SE = 0.217) | < .001 |
| Distance × Face age (RTs) | b | – | 5.363 (SE = 0.269) | < .001 |
| Distance × Face sex (RTs) | b | – | 1.547 (SE = 0.192) | < .001 |
| Distance × Face age × Face sex (RTs) | b | – | 0.654 (SE = 0.274) | .016 |

**Experiment 2**

**
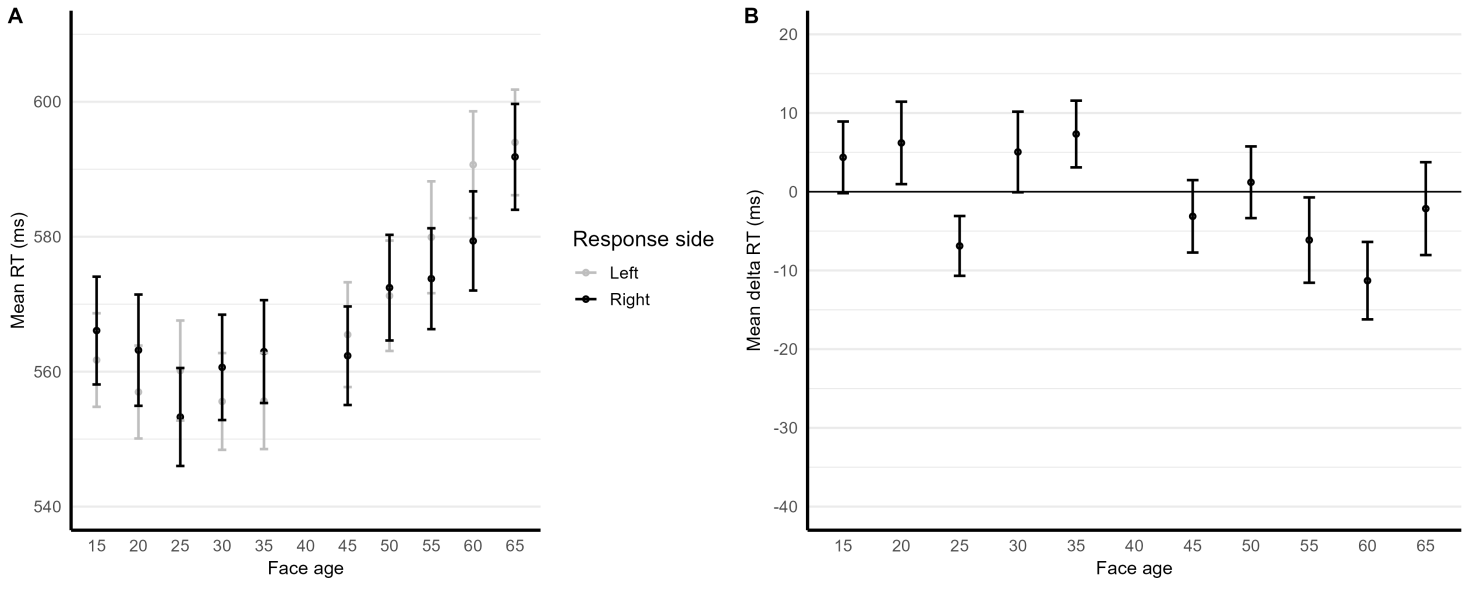
**

**Figure S2**. Panel A shows mean RTs, averaged across participants, as a function of face age and response side. RTs do not vary as a simple monotonic function of face age. Panel B shows the mean difference between RTs for the right and left response keys (delta RT), averaged across participants, as a function of face age. Delta RT likewise tends to exhibit a step-like rather than continuous pattern. Overall, the patterns observed in Panels A and B justify treating face age as a dichotomous (younger vs. older), rather than continuous, predictor in the main STEARC analysis. In both panels, error bars represent the standard error of the mean.

*2A*

The analysis revealed significant main effects of face age, *χ*^2^(1) = 37.08, *p* < .001, with longer RTs for older faces (*M* = 590 ms, *SE* = 2.36) than for younger faces (*M* = 573 ms, *SE* = 2.02); face sex, *χ*^2^(1) = 7.62, *p* = .006, with longer RTs for female faces (*M* = 586 ms, *SE* = 2.44) than for male faces (*M* = 577 ms, *SE* = 2.38); and participant sex, *χ*^2^(1) = 118.43, *p* < .001, with longer RTs for male participants (*M* = 605 ms, *SE* = 2.66) than for female participants (*M* = 558 ms, *SE* = 2.77). Response side was non-significant, *χ*^2^(1) = 0.002, *p* = .958. A significant face age × face sex interaction also emerged, *χ*^2^(1) = 18.93, *p* < .001. Further comparisons showed that, for older faces, the RTs were longer (*p* < .001) for female faces (*M* = 604 ms, *SE* = 3.69) than for male faces (*M*= 577 ms, *SE* = 3.63), while for younger faces the opposite non-significant trend emerged, with longer RTs (*p* = .122) for male faces (*M*= 577 ms, *SE* = 3.21) than for female faces (*M*= 569 ms, *SE* = 3.33).

*2B*

As recommended by one reviewer, we also conducted exploratory analyses restricted to right-handed participants. The main results replicated those obtained from the full sample (see the analysis file on OSF). Concerning the main hypothesis, the analysis confirmed that, for older faces, RTs were longer with the left-side key (*M* = 595 ms, *SE* = 2.51) than with the right-side key (*M* = 591 ms, *SE* = 2.56), whereas for younger faces RTs were longer with the right-side key (*M* = 574 ms, *SE* = 2.79) than with the left-side key (*M* = 573 ms, *SE* = 2.69). However, the response side × face age interaction was no longer significant, *χ*²(1) = 2.55, *p* = .110, likely reflecting reduced statistical power due to the approximately 20% decrease in sample size.

*2C*

As recommended by one reviewer, the same pattern of interactions also emerged in the exploratory analysis restricted to right-handed participants (see the analysis file on OSF).

*2D*

The analysis revealed significant main effects of face age, *χ*^2^(1) = 20.89, *p* < .001, with higher accuracy for younger faces (*M* = 0.983, *SE* = 0.002) than for older faces (*M* = 0.969, *SE* = 0.003); response side, *χ*^2^(1) = 9.32, *p* = .002, with higher accuracy for responses with the right-side key (*M* = 0.979, *SE* = 0.002) than the left-side key (*M* = 0.975, *SE* = 0.003); and face sex, *χ*^2^(1) = 6.10, *p* = .013, with higher accuracy for male faces (*M* = 0.980, *SE* = 0.002) than for female faces (*M* = 0.974, *SE* = 0.003). Participant sex was non-significant, *χ*^2^(1) = 1.15, *p* = .282. Analyses restricted to right-handed participants yielded results similar to those obtained from the full sample, with the following exceptions. The main effect of face sex was not significant, *χ*²(1) = 1.72, *p* = .190. A significant face age × face sex interaction emerged, *χ*²(1) = 4.17, *p* = .041. Further comparisons indicated that, for older faces, accuracy was higher for male (*M* = 0.977, *SE* = 0.003) than for female faces (*M* = 0.971, *SE* = 0.005), *p* = .014, whereas for younger faces, accuracy did not differ between face sexes, *p* = .616. A significant response side × face sex interaction also emerged, *χ*²(1) = 7.20, *p* = .007. Further comparisons showed that, for female faces, accuracy was higher with the right-side key (*M* = 0.982, *SE* = 0.003) than with the left-side key (*M* = 0.970, *SE* = 0.004), *p* < .001, whereas for male faces, accuracies for the left and right keys did not differ, *p* = .415.

*2E*

For younger faces, the best-fitting model included distance and face sex as fixed effects, and by-participant intercepts and slopes for face sex as random effects. The effect of distance (*b* = 0.157, *SE* = 0.158, *p* = .21) and the interaction between distance and face sex (*b* = 0.075, *SE* = 0.20, *p* = .706) were non-significant. For older faces, the best-fitting model included distance and face sex as fixed effects, and by-participant intercepts and slopes for face sex as random effects. The interaction between distance and face sex was significant (*b* = 1.391, *SE* = 0.210, *p* < .001), reflecting a stronger inverted distance effect for female faces (*b* = 2.162, *SE* = 0.169, *p* < .001) than for male faces (*b* = 0.771, *SE* = 0.163, *p* < .001).

**Table 2.** Main results observed in Experiment 2.

| *Effect* | *Test* | *df* | *Statistic* | *p* |
| --- | --- | --- | --- | --- |
| Response side × Face age (RTs) | χ² | 1 | 4.43 | .035 |
| Response side × Face age × Face sex (RTs) | χ² | 1 | 0.01 | .756 |
| Response side × Face age × Participant sex (RTs) | χ² | 1 | 0.87 | .351 |
| Response side × Face age × Face sex × Participant sex (RTs) | χ² | 1 | ≈ 0.0 | .979 |
| Response side × Face sex (RTs) | χ² | 1 | 11.45 | < .001 |
| Response side × Face sex × Participant sex (RTs) | χ² | 1 | 11.16 | < .001 |
| Response side × Face age (Accuracy) | χ² | 1 | 2.34 | .126 |
| Distance (RTs) | b | – | 2.371 (SE = 0.196) | < .001 |
| Distance × Face age (RTs) | b | – | 2.206 (SE = 0.194) | < .001 |
| Distance × Face sex (RTs) | b | – | 1.571 (SE = 0.225) | < .001 |
| Distance × Face age × Face sex (RTs) | b | – | 7.273 (SE = 1.431) | < .001 |

**Reference**

Casasanto, D. (2009). Embodiment of abstract concepts: good and bad in right-and left-handers. *Journal of Experimental Psychology: General*, *138*(3), 351. https://doi.org/10.1037/a0015854
